# A Cloud-Based System for Automated AI Image Analysis and Reporting

**DOI:** 10.1007/s10278-024-01200-z

**Published:** 2024-07-31

**Authors:** Neil Chatterjee, Jeffrey Duda, James Gee, Ameena Elahi, Kristen Martin, Van Doan, Hannah Liu, Matthew Maclean, Daniel Rader, Arijitt Borthakur, Charles Kahn, Hersh Sagreiya, Walter Witschey

**Affiliations:** 1 Department of Radiology, University of Pennsylvania, Philadelphia, USA; 2 Perlman School of Medicine, University of Pennsylvania, Philadelphia, USA; 3 Department of Information Services, University of Pennsylvania, Philadelphia, USA; 4 Department of Bioengineering, University of Pennsylvania, Philadelphia, USA; 5 Department of Medicine, University of Pennsylvania, Philadelphia, USA; 6 Department of Radiology, Northwestern University Feinberg School of Medicine, Chicago, USA

**Keywords:** Informatics, AI, Opportunistic screening, Steatosis, AI orchestrator

## Abstract

Although numerous AI algorithms have been published, the relatively small number of algorithms used clinically is partly due to the difficulty of implementing AI seamlessly into the clinical workflow for radiologists and for their healthcare enterprise. The authors developed an AI orchestrator to facilitate the deployment and use of AI tools in a large multi-site university healthcare system and used it to conduct opportunistic screening for hepatic steatosis. During the 60-day study period, 991 abdominal CTs were processed at multiple different physical locations with an average turnaround time of 2.8 min. Quality control images and AI results were fully integrated into the existing clinical workflow. All input into and output from the server was in standardized data formats. The authors describe the methodology in detail; this framework can be adapted to integrate any clinical AI algorithm.

**Supplementary Information:**

The online version contains supplementary material available at 10.1007/s10278-024-01200-z.

## Introduction

The rapid development of academic and commercial artificial intelligence (AI) algorithms has exploded in recent years, but clinical adoption has not kept pace with the rate at which AI algorithms are being released. As of December 6, 2023, the U.S. Food and Drug Administration (FDA) had cleared 692 AI algorithms for clinical usage, of which 531 were cleared for radiology [1]. Yet a major barrier towards AI adoption is seamless integration into the clinical workflow. Hence, there is a need to describe the successful integration of algorithms, evaluate their use, demonstrate their value to the practice of radiology, and encourage their adoption.

AI has demonstrated many theoretical benefits to radiology practice. Research has suggested that AI has the potential to help radiologists avoid missed diagnoses, increase workflow efficiency, reduce burnout, and improve diagnostic accuracy [2–5]. It may allow for easier longitudinal monitoring and may enable increased opportunistic screening [6, 7]. There are some successful examples showcasing how AI can be integrated into a real clinical workflow, such as the RSNA Imaging AI in Practice (IAIP) demonstration [8] and an AI tool to create lymphoscintigraphy reports [9].

There are several factors that have made seamless integration of algorithms into clinical workflows challenging. These challenges and their solutions can be identified by AI governance and management structures embedded in the organization [10]. Its members include stakeholders from radiology, information technology (IT), and clinical practitioners. Radiologists desire AI to be fast, unobtrusive (well integrated with radiology infrastructure), and explainable. IT stakeholders desire AI that is secure, standardized, and centralized. However, commercially available AI solutions are frequently siloed and do not seamlessly integrate with the wider enterprise software ecosystem and workflows.

To meet this need, we developed an AI orchestrator [8] integrated with an AI tool for an opportunistic screening application with seamless PACS and reporting engine integration. As a proof-of-concept, we developed an AI algorithm to screen for and report hepatic steatosis and deployed it in a live clinical environment. Since CT is commonly performed for reasons other than to assess liver disease, missed diagnoses of hepatic steatosis can occur frequently [11] and a large majority of patients are unaware they have the disease [12–14]. However, the manual extraction of imaging traits for hepatic steatosis can be burdensome in a high-volume practice. To address this issue, previously developed and validated methods using deep learning were used to segment the liver [15, 16]. Given the volume of imaging studies and the estimated 32% prevalence of hepatic steatosis in the global population(17), it was anticipated that the AI workflow with an application for hepatic steatosis would be able to identify moderate-to-severe disease in a large proportion of patients.

## Materials and Methods

### IRB

The project was reviewed by our Institutional Review Board. It was determined that the project met eligibility criteria for IRB review exemption due to a retrospective review of data and deidentification of patient identifying information. The study was performed in accordance with the ethical standards as laid down in the 1964 Declaration of Helsinki and its later amendments.

### Health System and Governance

The project was performed at a multi-site health system that consisted of a US-based urban tertiary care center and multiple satellite hospitals and outpatient centers with a regional care distribution. The need for the AI orchestrator to be deployed across the multiple hospitals and centers required a governance committee to be established. The hospital system established a governance structure consisting of faculty and staff from radiology and the information technology departments (Clinical AI Steering Committee). These personnel included clinical radiologists and IT personnel and were under the authority of the chair of the radiology department and the chief information officer of the health system.

Governance elected to move forward with the AI orchestrator development and pilot application targeting opportunistic screening of hepatic steatosis. The project team personnel included clinical radiologists, radiology researchers, business administrators, IT managers, and IT application analysts [18]. Since initiating the project, the project team has been continuously involved in the implementation, maintenance, and monitoring of the clinical AI algorithms. Team members met weekly to determine project goals, set milestones, and evaluate progress [16].

### Stakeholder-Based Assessment of Needs and Software Requirements

The initial task of the project members was to conduct stakeholder-based needs assessment to determine the software and hardware requirements of the health system. The project identified multiple sets of stakeholders including radiologists, IT personnel, patients, and primary caregivers. Initial needs were prioritized around radiologist and IT requirements to assure that a clinical workflow could be supported in the pilot application.

From the perspective of radiologist stakeholders, there was a strong need for the AI findings to be seamlessly integrated with the clinical workflow. Because of the increasing annual volume of radiologic imaging services, it was critical that an AI workflow did not add significant additional time to the reading of the study. Thus, the AI results needed to be available by the time the radiologist reviewed the study during their normal clinical workflow. This time constraint was initially defined as a period of 10 min from the finalization of the imaging study to when complete AI findings were available. The system also needed to be unobtrusive, with minimal interaction to limit interruptions to normal workflow. Finally, verification of the results was a requirement with intuitive quality control that the radiologist can review before including results into a report.

Separate from the needs of the radiologist, there were enterprise requirements defined by the IT stakeholders. Data security had the highest priority with patient data protected within the IT network of the health system with no outside unencrypted internet traffic. The tool needed to be integrated across multiple sites at physically distinct locations and integrate with different hardware and software enterprise platforms already in use in the health system. There would be full adherence to standards for medical imaging, including output and communication between different components of the enterprise system using standardized software file structures and protocols such as DICOM and HL7 [19, 20]. There was also a need to scale the system based on the demand for imaging services, while being affordable with the ability to potentially expand to other platforms and applications.

### Summary of the Clinical AI Workflow

**Fig. 1:**
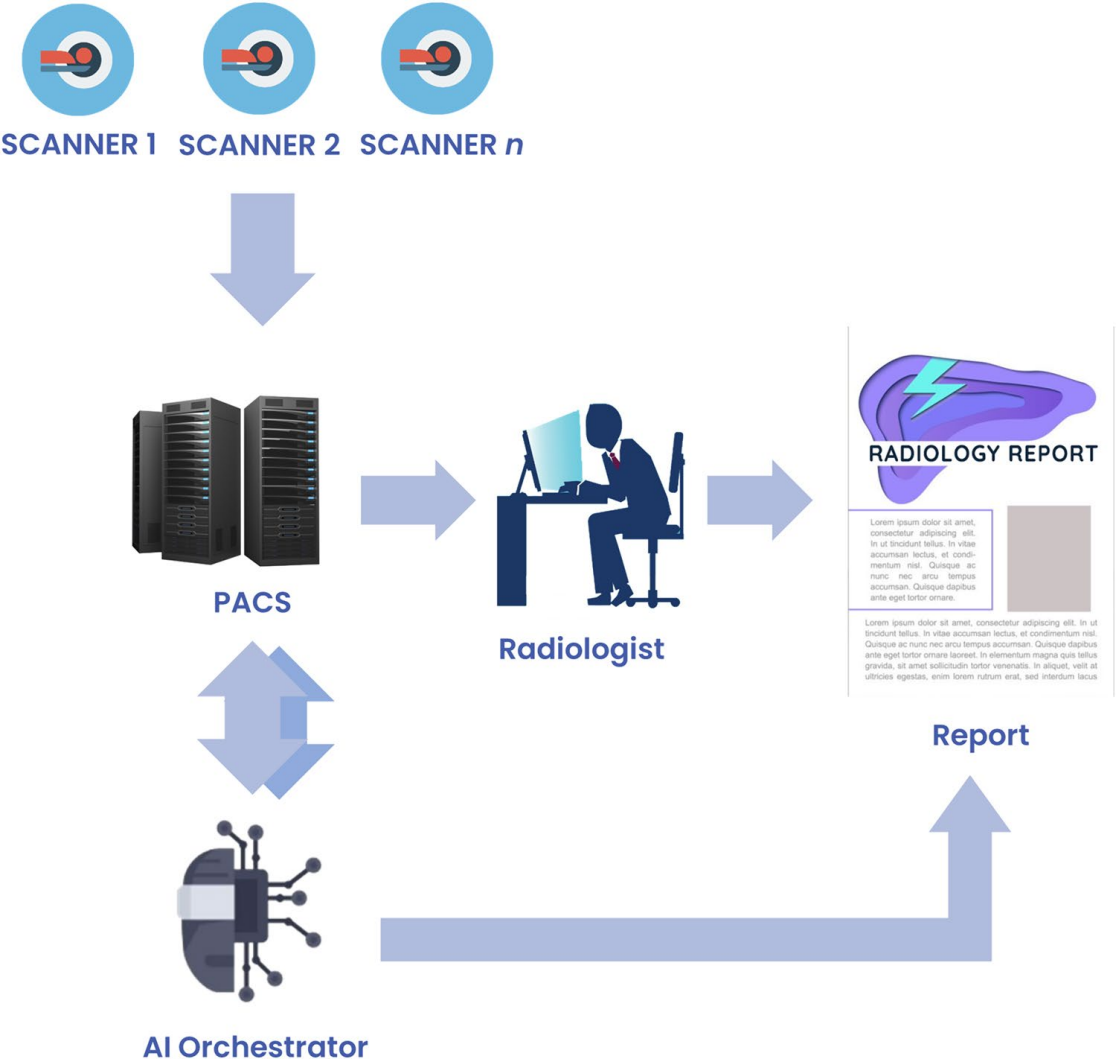
Broad overview of AI orchestrator implementation. The AI orchestrator exchanges data with the PACS system, including AI-generated quality control images for radiologist review, and AI results are sent directly to the radiology report

An overview of the software architecture is shown in Fig. 1. The key steps for AI processing are summarized here, and each individual component is described in further detail in the following section. In the first step, CT scanners across the enterprise send studies to PACS. Upon completion of the study by the CT technologist, the PACS system transfers the study to a cloud-based AI Server. The server evaluates the images and determines the appropriate AI algorithms, if any, to run for each series within the study. The AI algorithms generate image overlays and summary results in the form of common data elements (CDE) [21, 22] packaged into DICOM structured reports (DICOM SR). Image overlays are appended to the study in PACS and reviewed by the radiologist for quality control. Finally, DICOM SRs are transferred to the reporting engine via an HL7 router and inserted into the report using a dictation macro.

### Detailed Description of the Clinical AI Workflow

**Fig. 2:**
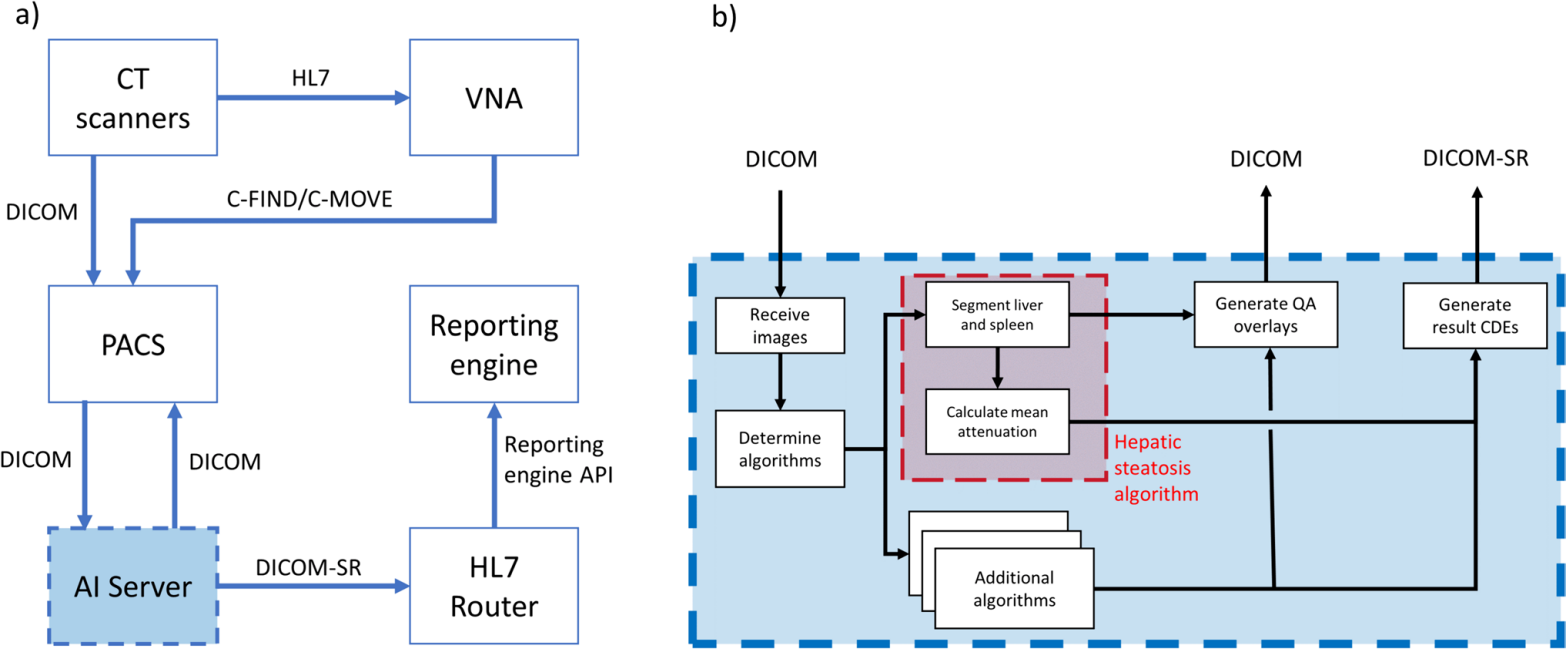
**a** Overview of the enterprise radiology system with AI server integration. **b** Detailed view of operations performed by the AI orchestrator housed on the AI server, with the example hepatic steatosis AI algorithm. All information flow into and out of the server uses standardized formats

A more detailed description of the clinical AI workflow is shown in Fig. 2, and the key components are described in this section.

#### Imaging Modalities

Clinical CTs acquired from multiple different physical locations within our healthcare system were included in this study. A summary of the locations and CT scanners included in this study is provided in Supplementary Data Table 1. The CT scanners are multivendor systems and include legacy and dual-energy CT (DECT) scanners using dual-source, kVp-switching, dual-layer, dual-source CTs (Somatom Definition Drive, Flash & Force, Siemens Healthineers), rapid kVp-switching CT (Discovery CT750 HD, GE Healthcare), and a dual-layer CT with 8-cm coverage (iQon 7500 Spectral CT, Philips Healthcare).

**Table 1:** Example section of the clinical report using AI measurements

Template	Sample report
ATTENUATION MEASUREMENTS: Liver ([RDE1194] HU) - spleen ([RDE1207] HU) = [RDE1193] HU Hepatic steatosis: [yes/no] Threshold: liver < 40 HU or liver-spleen < − 10 HU (Hamer O., et al. RadioGraphics 2006)	ATTENUATION MEASUREMENTS: Liver (30 HU) - Spleen (49 HU) = − 19 HU Hepatic steatosis: present [LIVF1] Threshold: liver < 40 HU or Liver-Spleen < -10 HU (Hamer O, et al. RadioGraphics 2006)

[RDE1194], [RDE1207] and [RDE1193] are common data elements (CDEs) in the reporting engine template. The AI Server sends the values for CDEs to the reporting engine, and these fields are automatically populated when the template is included in the report

#### PACS/VNA

CT data was sent to the centralized PACS (IDS7, Sectra AB, Linköping, Sweden) and image archive (Mach7, Mach7 Technologies, South Burlington, VT) systems in standard DICOM formats. After a CT study was marked as completed by a technologist (transferring an HL7 message from the EHR (EPIC) to Mach7), studies were automatically identified by the VNA for further AI processing if they satisfied Current Procedural Terminology (CPT) codes 74,176 (CT abdomen and pelvis without contrast) and 74150 (CT abdomen without contrast). The VNA automatically performs basic filtering on select DICOM fields to identify potential series-level data for AI analysis (for example, including source axial images but disregarding various reformats). This filtering step was done to reduce bandwidth requirements by omitting redundant/irrelevant data transfer. The VNA then sent an HL7 message to the PACS system, prompting it to transfer the appropriate study data to the AI server.

#### AI Server

AI orchestrator software was hosted on a cloud-based server (Microsoft Azure). To avoid data transfer over public networks, a virtual private network connection was established between the cloud server and the local intranet (Azure ExpressRoute). The virtual machine was a burstable system with 8 virtual CPUs, 32 GB RAM, and premium SSD LRS storage on a 3 TB disk. Imaging data was removed automatically from the storage after processing and a 1-week delay period.

#### AI Orchestrator

The AI orchestrator was a collection of software applications deployed on the AI server. The AI orchestrator included the following components. There was a DICOM sender and receiver to transfer DICOM images between the server and the PACS/VNA systems. There was an application with logic to determine which AI applications to run and what series to run them on. This application is flexible and can use any arbitrary data to select AI applications, which typically will involve a combination of study metadata and technical parameters for specific image series. For our example application of opportunistic hepatic steatosis screening, the orchestrator checked that [1] the study had an appropriate CPT code indicating that it was a non-contrast study, [2] evaluated the images to verify that it was a non-contrast study before running the hepatic steatosis algorithm and [3] checked that the study had an axial series that met technical inclusion criteria including a slice thickness within specified parameters (to save on processing time) and a uniform slice thickness. Software subsequently packaged results into CDEs and DICOM-SRs for exporting to the reporting engine and transmitted results and quality control images into DICOMs for exporting to PACS. Finally, logging software was developed to monitor system throughput and speed and to aggregate AI application results for research and quality control. Software was developed in Python (Python v. 3.11) using additional software libraries (Pynetdicom v. 3, pydicom, ITK, pandas).

#### Quality Control

**Fig. 3:**
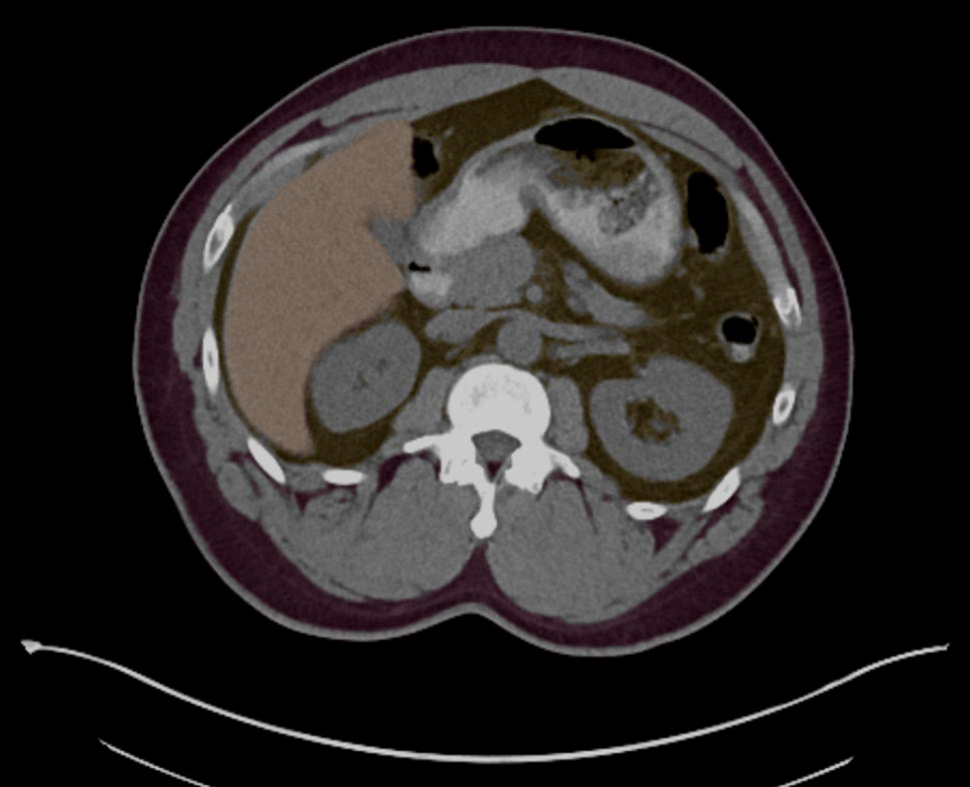
Sample quality control image sent to PACS from the AI Server. Transparent overlays show segmented liver, spleen, visceral fat, and subcutaneous fat from an AI segmentation algorithm used to screen for hepatic steatosis. This allows the radiologist to quickly verify accurate liver and spleen segmentations before including AI spleen and liver measurements in the clinical report

Quality control images were automatically generated by the AI orchestrator, packaged into a standard (DICOM) format, and exported to PACS for radiologist review during their normal clinical workflow. For our example application of opportunistic hepatic steatosis screening, image series were created showing liver and spleen segmentations overlaid on CT images so the radiologist can quickly verify AI segmentation accuracy. An example is shown in Fig. 3. An online form was also created to collect user feedback. Radiologists could use this form to leave feedback on algorithm performance or any other issues to be reviewed by the development team. Additionally, we hold weekly meetings including personnel from information services, research, and clinical radiology where concerns can be raised and immediately discussed.

#### Reporting Engine Integration

After AI algorithms finished running on the AI server, the AI orchestrator packaged summary AI results for the reporting engine. In our example application of hepatic steatosis screening, these results included mean attenuation in Hounsfield Units for the liver and spleen. All exported values had a designated CDE, and these CDEs were packaged into a DICOM-SR. A full list of CDEs used is listed in Supplementary Data Table 2. The DICOM-SR was then exported to the reporting engine (PowerScribe 360, Nuance Communications) via an HL7 router (Laurel Bridge, Laurel Bridge Software). CDEs could be accessed by using custom fields in the reporting template. We created a custom template for our example application that included mean liver and spleen attenuation (Table 1). To have AI results in their report, the radiologist only needed to invoke the macro to include the template, and the custom fields would populate automatically.

#### Database

After an AI algorithm was run for a study, the AI orchestrator appended all generated CDEs to a running database that could be used for quality control or research purposes. Detailed logs of file transfers and AI algorithm processing times were also kept for quality control.

### Hepatic Steatosis Assessment

As proof of concept, we deployed a fully automatic AI algorithm that screens for hepatic steatosis [15, 16]. Hepatic steatosis opportunistic screening was considered a low-risk test case for AI deployment as it was in most cases unrelated to the clinical indication for abdominal CT and radiologists were not dependent on AI results to provide care. The Clinical AI Steering Committee selected this as a good initial AI application. In brief, the algorithm fully segments the liver and spleen and calculates the mean attenuation in Hounsfield Units of each organ. The specific liver and spleen segmentation algorithm was chosen based on prior experience with the algorithm, and details of algorithm development and optimization are described in detail in the original manuscripts. The difference between mean liver HU and mean spleen HU was then used to evaluate for hepatic steatosis [23, 24]. We report the spleen-hepatic attenuation difference (SHAD). SHAD > 10 and mean hepatic attenuation < 40 HU were used as the radiologic determinant for moderate to severe hepatic steatosis [25, 26].

### AI Orchestrator and AI Server Performance Analysis

Detailed logs of all AI orchestrator actions were kept for analysis. For each study, logging included the exact time that each DICOM was received or sent and the exact start and stop time for each algorithm that was run.

Turnaround time (TAT) was defined as the time elapsed from receipt of the first DICOM image to the successful transfer of all quality control images to PACS and results (in the form of DICOM-SRs) to the reporting engine. TAT was further broken down into two separate components: [1] study transfer time, which is the time to transfer a study from PACS to the AI server (defined as time elapsed between the receipt of the first and last DICOM from the study) and [2] AI processing time, which is the time to run AI algorithms and export quality control image results from the AI server to the PACS and reporting engine (defined as time between the receipt of the last DICOM from PACS and the transfer of all DICOMs and DICOM-SRs to PACS and the reporting engine). In practice, transferring results out of the AI server took a negligible amount of time, and the AI processing time almost entirely consists of the time spent running AI algorithms.

## Results

### AI Orchestrator and AI Server Performance

**Fig. 4:**
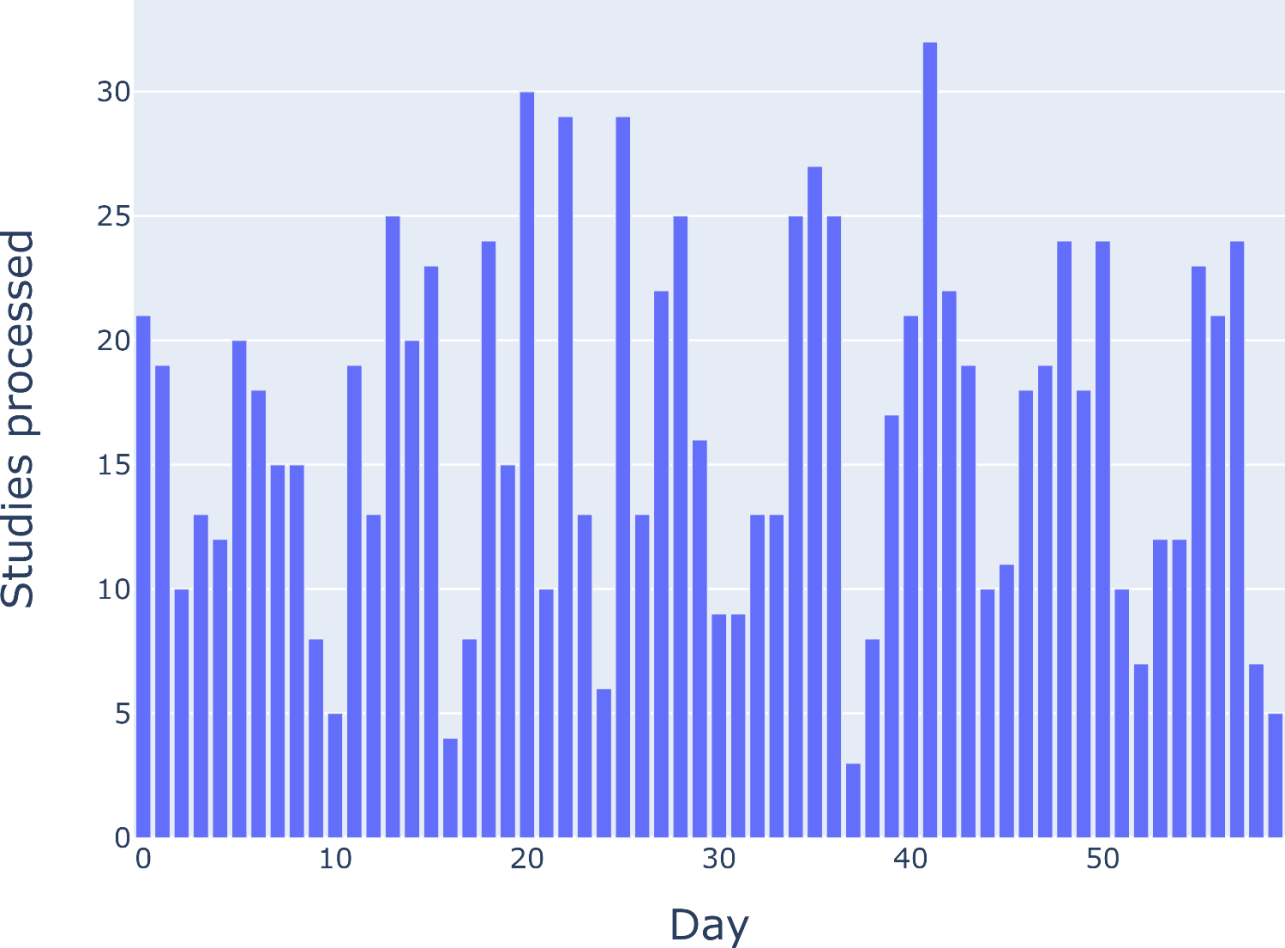
Number of non-contrast CTs processed per day over a 60-day period. All non-contrast abdominal CTs at multiple separate physical sites were sent to the AI server for opportunistic hepatic steatosis screening

**Fig. 5:**
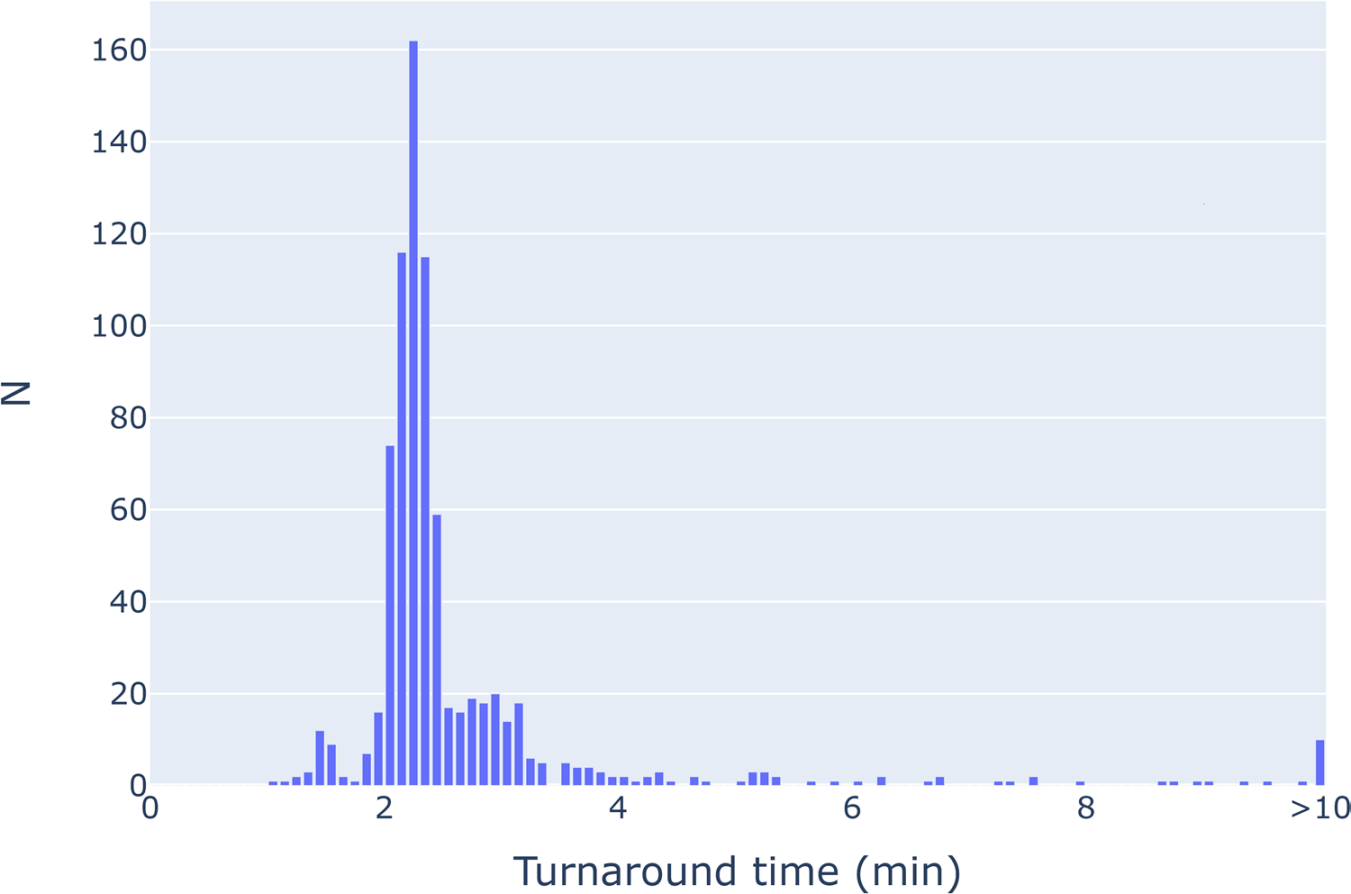
Distribution of turnaround times (TAT) in a 60-day period. TAT is defined as the time from the start of the study transfer to the AI server to the completion of exporting AI results to PACS and the reporting engine. The average TAT was 2.8 min, and 94.9% of studies had a TAT of < 5 min

In a 60-day period, 991 studies were processed (Fig. 4). The average AI processing time was 1.4 min, the average transfer time was 1.5 min, and the average TAT was 2.8 min. 94.9% of studies had a TAT of less than 5 min. The maximum TAT was 46.5 min. These results are shown in more detail in Fig. 5.

### Study Throughput and Error Analysis

An independent search of our clinical reporting database identified 3178 studies over the same 60-day period that had CPT codes indicating they were potentially eligible for AI processing. 2697 studies (85%) were transferred to the server. Prior to starting AI processing, the AI orchestrator checked to see if the study met technical inclusion criteria for evaluation (slice thickness within specified range and slick thickness uniformity). One thousand seven hundred six studies (63%) were rejected for not meeting technical inclusion criteria and 991 studies (37%) were selected for AI analysis. The AI analysis pipeline ran to completion on 991 studies (100%). Results were successfully transferred to the PACS and reporting engine for 784 studies (79%). Subsequent investigation showed that unsuccessful transfers were due to network connection errors and that failed transfers occurred in batches. This is summarized in Supplementary Fig. 1.

### Cost

There were two major contributors to cost: clouding computing infrastructure and personnel to develop and maintain the system.

#### Cloud Computing

Compute and storage costs for our virtual machine were approximately $5 k per 10,000 studies over a 1-year period (including additional studies beyond the 60-day period analyzed in detail for this manuscript). Exact prices varied based on the need for additional temporary storage or compute capabilities. A graph of price per month is shown in Supplementary Fig. 2.

#### Personnel

The cost of the initial development of the platform included 6 calendar months of a full-time software engineer with experience in the DICOM protocol, networking, and implementing previously developed AI modules. Deployment and maintenance of this platform also required an onsite information services team with experience in PACS/VNA management and cloud computing. This required approximately 0.6–1.2 calendar months for initial setup and approximately 0.3 calendar months per year for subsequent maintenance.

### Hepatic Steatosis Opportunistic Screening

**Fig. 6:**
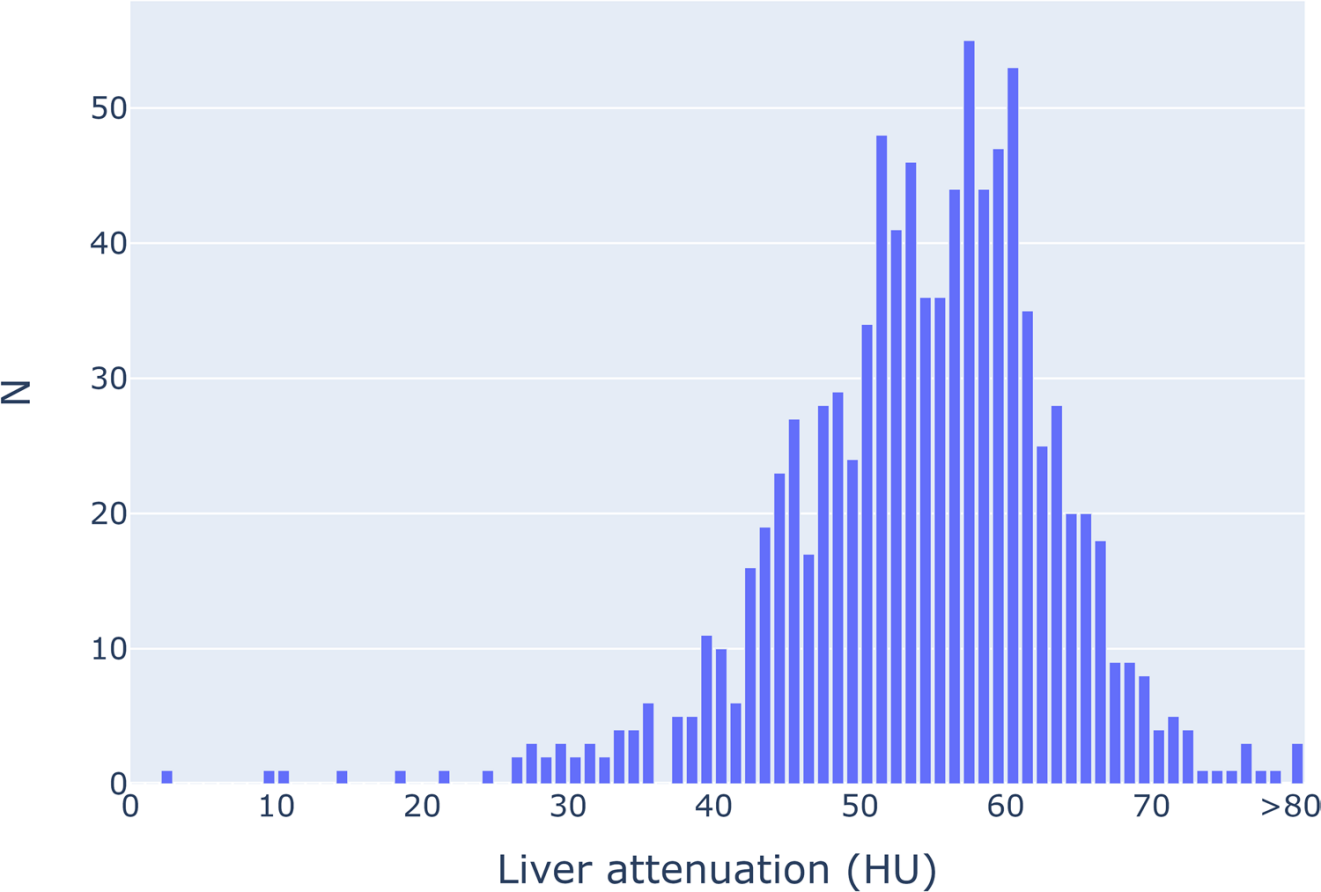
Distribution of hepatic attenuation of non-contrast abdominal CTs performed over a 60-day period. Attenuation of < 40 HU is consistent with hepatic steatosis

Results from hepatic steatosis opportunistic screening are shown in Fig. 6. The mean SHAD and hepatic attenuation were − 11.9 and 54.3 HU respectively. Hepatic steatosis prevalence was 2.2% using a cutoff of SHAD > 10 and 6.4% using a cutoff of hepatic attenuation < 40. The average hepatic volume was 1529 mL.

## Discussion

We adopted a shared governance model of AI oversight and identified the AI needs of radiologists and the larger IT/enterprise infrastructure that they are a part of. We implemented a cloud-based AI orchestrator in a multi-hospital health system that met both AI and IT/enterprise needs. As a proof of concept, we used AI to opportunistically screen for hepatic steatosis on every non-contrast abdominal CT in our healthcare system. In 60 days, we processed 991 studies across multiple separate physical sites with an average turnaround time of 2.8 min.

Radiologist AI needs focused on the day-to-day workflow: AI must seamlessly integrate into the software platforms they already use and cause minimal interruption to their work. Meanwhile, IT/enterprise AI needs are focused on seamless AI integration into the larger enterprise software ecosystem in a way that is secure, maintainable, and scalable.

These needs are not mutually exclusive, but they are different, and AI that works for one group does not necessarily work for the other. A common example of this a “siloed” commercial solution that performs well from a radiologist perspective but requires its own onsite hardware or sends data offsite for processing, which can create systemic complexity and security concerns from an enterprise perspective.

We designed a system to meet both radiologist and enterprise needs by adopting an AI orchestrator framework [8]. In short, AI becomes another system component, analogous to the PACS, VNA, EMR, etc. This has been successfully developed and implemented before in an academic setting in a limited scope for processing lymphoscintigraphy data [9]. Commercial AI orchestrators are now also starting to become available as well. These would be an attractive solution for many radiology enterprises looking to incorporate AI into their workflow, especially smaller institutions or practices that are not able to or do not want to spend internal IT resources on developing and maintaining a custom internal solution.

The compute and storage costs for our system were approximately $450/month for storage and $250/month for compute time, though these costs depend greatly on the type and resilience of storage and compute. Beyond compute and storage, a significant cost for most organizations is likely personnel for the development of new software, deployment, and maintenance. Even if development can be minimized, such as by using an open-source solution, there is cost associated with the effort needed from experienced information services personnel for deployment (approximately 0.6–1.2 calendar months) and maintenance (0.3 calendar months per year). To determine whether this is more costly than a commercial solution requires weighing these costs against the savings gained from avoiding software licensing fees, which in practice will strongly depend on an organization’s IT environment and resources. However, independent of cost, using a custom internal solution such as the one in [9] or the one presented here offers a level of flexibility, transparency, and data control that may be difficult to achieve with a commercial solution. An open-source AI orchestrator solution may be particularly beneficial in academic hospitals, which would use the orchestrator to deploy novel AI technologies quickly. Underserved communities would also benefit from open-source solutions due the limited resources available to them to support enterprise solutions.

Here we demonstrate a successful custom implementation at a larger scale: processing non-contrast CTs of the abdomen across 3 separate hospitals, which in a 60-day period amounted to 988 studies. Our calculated moderate to severe steatosis prevalence of 6.0% closely aligned with a previously reported prevalence of 6.2% [25], and our calculated mean volume of 1529 mL is similar to previously reported volumes ranging from 1240 to 1520 mL [27–29].

Our AI orchestrator was built with enterprise AI needs in mind. We implemented our AI orchestrator downstream from our centralized PACS, which makes it inherently agnostic to the make, model, or physical location of any modality that is sending data to the PACS. All information that flows into and out of the orchestrator is in standardized DICOM and DICOM-SR formats. This enables easier integration into the enterprise software ecosystem and allows for greater future system flexibility (for example, upgrading or changing the PACS or the reporting engine).

Data security is critical from an enterprise point of view. As of December 20, 2023, there were 349 separate data breaches at healthcare providers affecting at least 500 people that were reported [30]. A cloud-based AI orchestrator gives us the ability to scale at a reasonable cost while maintaining data security. While we did transfer data to a server hosted by a third party (Microsoft Azure), we did so by using a private connection so that no data is transferred over the public internet, we fully controlled the software running on the server, and no data was transferred outside of our organization’s internal network. We viewed this as acceptable given the cost and scaling afforded by using a cloud-based implementation. We estimated our monthly server costs as only $450/month, and this could be decreased further with additional hardware and software optimization. It is also straightforward to add more storage and processing power as clinical volume increases and additional AI algorithms are deployed.

Radiologists need AI that is fast, unobtrusive, and verifiable, and we were able to implement AI that met those needs. Our average TAT was 2.8 min, and TAT was < 5 min 94.9% of the time, so for the vast majority of non-emergent cases AI results are ready by the time the radiologist opens the study. We kept AI unobtrusive by fully integrating results and quality control images into our existing clinical workflow so that using AI did not require switching applications or excessive extra clicks. Including quality control images natively in our PACS also lets our radiologists easily verify the accuracy of AI output.

Our AI orchestrator is not without limitations. It is important to recognize that the orchestrator is only a platform for the deployment of AI algorithms, and it does not solve any inherent limitations of the AI algorithms themselves. In particular, the successful technical deployment of an algorithm is not a guarantee of algorithmic accuracy, and AI results provided by the orchestrator are only as good as the underlying AI algorithms themselves. Known problems with AI algorithms such as model drift can still arise, and identifying and correcting for these problems requires continuous and robust oversight and monitoring. For example, the AI orchestrator is designed to easily incorporate new scanner locations and hardware into the AI processing pipeline, but the successful technical deployment of an AI algorithm at a new site does not guarantee AI results at the new site are of the same quality. Ensuring the same quality at a new site would require independent vetting of AI results from the new site, for example, by a visual check of AI segmentation results or by comparison to gold standard radiologist segmentations. Our AI orchestrator is built with these limitations in mind, and incorporating quality control images directly into the clinical workflow allows for radiologists to vet AI results at an individual patient level before including them in clinical reports. But additional ongoing monitoring is required to evaluate for and promptly address AI model drift and bias. Ideally, this would involve regular monitoring of AI results, both at a randomized sampling at the individual level (e.g., verifying organ segmentation) and by tracking aggregate results over time (e.g., monitoring population level drifts in steatosis).

There are opportunities to further improve efficiency, improve reliability, and reduce costs. While the TAT of 2.8 min was adequate for most studies, for truly time-critical cases, such as trauma or stroke, this may be too slow. The successful processing rate for studies that were sent to the AI server and met technical criteria for AI analysis was high. However, some processed results were not exported in real time to PACS and the reporting engine over the 2-month period analyzed. Subsequent analysis showed this to be due to issues with network connectivity during long system uptimes of more than a week. To address this, we have since implemented a schedule for an automated reset of the AI server. Additional approaches that we have begun implementing to improve resilience are to provide automated alerts to personnel, AI monitoring tools (dashboarding), and implementing redundant connections and failover mechanisms. Additionally, even if studies were transmitted to the AI server because they had appropriate billing codes for processing, many were not processed as images were found to have parameters that were not tested during the initial development of the segmentation algorithms (e.g., inconsistent slice thickness or non-axial field-of-view orientations). While loosening these restrictions may be appropriate to capture a greater percentage of the patient population for opportunistic screening, it may be necessary also to reevaluate the quality of AI annotations for datasets in which scan parameters lie outside the training domain.

Robust failure management will be necessary for many enterprise applications of AI orchestrators. Adopters of this technology should realize potential failure states in advance, and requirement analysis should be performed to determine the requirements and resilience of the software and assure they are aligned with the clinical and business objectives of the radiology practice. For instance, Gibney et al. apply Hazard, Risk, and Vulnerability analysis to determine the critical weaknesses of radiology departments [31]. Importantly, we established that opportunistic screening for liver disease was a low-risk application for developing a pilot AI orchestrator. For most studies, steatosis was unrelated to the clinical indication for abdominal CT, and failures were tolerated for this application as the radiologist was not dependent on the AI finding to provide care. Software programming errors can potentially be further mitigated by the adoption of open source and Findable, Accessible, Interoperable, and Reusable (FAIR) principles [32–34] by allowing for more developers and users to find and fix software issues. We expect that continuous improvements, experience, and providing open-source software to the community will help further improve resilience and further reduce the failure rate.

During development, failures of the system were encountered and expected. Monitoring systems will help identify errors as they emerge and provide automated alerts to users. We provided an online form that allowed radiologists to comment and provide feedback on potential failures, such as missing AI results. Additionally, study queuing was observed during initial stages of development. Queuing was observed when the compute infrastructure was unable to keep pace with an inflow of new imaging studies, resulting in a queue of studies waiting to be processed. In this pilot application, queuing was overcome by reducing the load on the server. In the initial stage of development, the load was reduced simply by eliminating thoracic CT studies from AI analysis. Beyond this, employing comprehensive integration, system, and user acceptance testing will be essential for many applications. Furthermore, there should be processes in place for personnel and software change management, along with documentation and knowledge management for users and developers.

There are important questions about ethical AI practices such as how to assure AI algorithms and orchestrators will respect human rights and freedoms, including dignity and privacy [35]. This is why it is critical that AI results are interpretable and dependable. For our pilot task, providing segmentation overlays to the radiologist helped provide transparency by allowing the radiologist to evaluate segmentation quality and reject results derived from flawed segmentations. The AI segmentation algorithms were developed from a disease-agnostic cohort of patients [15, 16]. However, it should be recognized that such clinical cohorts may have intrinsic biases associated with the underlying training set patients [36]. Additionally, cybersecurity is a concern that requires assuring privacy of patient data and preventing attacks, and we used data encryption to send information to the AI server. While images were sent to the server for analysis, they had a short 1-week lifecycle before deletion. The PACS system provided redundant storage of AI-enhanced results (image overlays and DICOM SRs containing quantitative findings).

Although we found that the $700/month in cloud computing costs were relatively small given the volume of studies processed over this period (< $2 per study), it will be important to monitor these expenses as additional algorithms are added and their complexity begins to increase. There is room to decrease costs by optimizing hardware and software, but ultimately the cost of implementing AI will need to be weighed against gains in efficiency and patient outcomes. In theory, using AI for opportunistic screening to identify disease processes such as hepatic steatosis should lead to earlier diagnosis by identifying these diseases when they might otherwise have been missed. Earlier diagnosis should in theory lead to earlier intervention and improved patient outcomes, and the FDA recently approved a medication for the treatment of non-alcoholic steatohepatitis to be used along with diet and exercise [37]. Yet more work is needed to determine whether these theoretical benefits translate into improved real-world patient outcomes, and there are important regulatory and reimbursement challenges. For example, more work is needed to investigate how many additional cases of steatosis are identified when AI screening supplements regular radiology reads and whether the benefits of earlier intervention outweigh the potential downside of overdiagnosis. In addition to the need for ethical and interpretable AI methods for screening, A Radiology Scientific Expert Panel [38] recognized that standardization of AI data elements and AI reporting are needed, along with age, sex, and ethnicity adjusted normative data for the screening application.

An exciting and often understated benefit of implementing an AI orchestrator is the huge amount of research data that can be accumulated. In addition to exporting results into the clinical workflow, it is easy to simultaneously export results into a research database. The passive accumulation of AI-processed clinical data can be aggregated easily into databases with hundreds of thousands of imaging studies. At the rate we processed non-contrast CTs for our proof of concept, we would accumulate more than 6000 hepatic steatosis screening exams in 1 year. When integrated with a medical biobank (39), these large, processed datasets have the potential to facilitate big data research on risk factors and patient outcomes.

## Conclusions

AI needs of radiologists and the larger IT/enterprise infrastructure they are a part of can be identified using a shared governance model. It is possible to meet these separate needs using a cloud-based AI orchestrator, and we demonstrated this by implementing an AI algorithm to opportunistically screen for hepatic steatosis on non-contrast abdominal CTs in a multi-site healthcare system. This platform can be expanded to increase AI adoption within our own healthcare system, and it provides a framework for how AI can be successfully implemented in other healthcare systems.

## Supplementary Information

Below is the link to the electronic supplementary material.

Supplementary file1 (TIF 224 KB)

Supplementary file2 (TIF 344 KB)

Supplementary file3 (DOCX 18.9 KB)

## Data Availability

De-identified data is available upon reasonable request to the authors.

## References

[1] Artificial intelligence and machine learning (AI/ML)-enabled medical devices. https://www.fda.gov/medical-devices/software-medical-device-samd/artificial-intelligence-and-machine-learning-aiml-enabled-medical-devices. 10.1001/jamapediatrics.2024.5437 PMC11791695 39680415

[2] Bates DDB, Pickhardt PJ. CT-derived body composition assessment as a prognostic tool in oncologic patients: From opportunistic research to artificial intelligence–based clinical implementation. American Journal of Roentgenology. 2022;219(4):671–680. 10.2214/AJR.22.27749. 35642760 10.2214/AJR.22.27749

[3] Rudie JD, Rauschecker AM, Xie L, et al. Subspecialty-level deep gray matter differential diagnoses with deep learning and Bayesian networks on clinical brain MRI: A pilot study. Radiol Artif Intell. 2020;2(5):e190146. 10.1148/ryai.2020190146. 33937838 10.1148/ryai.2020190146 PMC8082339

[4] Buls N, Watté N, Nieboer K, Ilsen B, de Mey J. Performance of an artificial intelligence tool with real-time clinical workflow integration – Detection of intracranial hemorrhage and pulmonary embolism. Physica Medica. 2021;83:154–160. 10.1016/j.ejmp.2021.03.015. 33774340 10.1016/j.ejmp.2021.03.015

[5] Shoshan Y, Bakalo R, Gilboa-Solomon F, et al. Artificial intelligence for reducing workload in breast cancer screening with digital breast tomosynthesis. Radiology. 2022;303(1):69–77. 10.1148/radiol.211105. 35040677 10.1148/radiol.211105

[6] Bera K, Braman N, Gupta A, Velcheti V, Madabhushi A. Predicting cancer outcomes with radiomics and artificial intelligence in radiology. Nat Rev Clin Oncol. 2022;19(2):132–146. 10.1038/s41571-021-00560-7. 34663898 10.1038/s41571-021-00560-7 PMC9034765

[7] Pickhardt PJ, Summers RM, Garrett JW, et al. Opportunistic screening: Radiology scientific expert panel. Radiology. 2023;307(5). 10.1148/radiol.222044. 10.1148/radiol.222044 PMC10315516 37219444

[8] Wiggins WF, Magudia K, Schmidt TMS, et al. Imaging AI in practice: A demonstration of future workflow using integration standards. Radiol Artif Intell. 2021;3(6). 10.1148/ryai.2021210152. 10.1148/ryai.2021210152 PMC8637229 34870224

[9] Juluru K, Shih H-H, Keshava Murthy KN, et al. Integrating Al algorithms into the clinical workflow. Radiol Artif Intell. 2021;3(6). 10.1148/ryai.2021210013. 10.1148/ryai.2021210013 PMC8637237 34870216

[10] Chae A, Yao MS, Sagreiya H, et al. Strategies for implementing machine learning algorithms in the clinical practice of radiology. Radiology. 2024;310(1). 10.1148/radiol.223170. 10.1148/radiol.223170 PMC10831483 38259208

[11] Wright AP, Desai AP, Bajpai S, King LY, Sahani D V, Corey KE. Gaps in recognition and evaluation of incidentally identified hepatic steatosis. Dig Dis Sci. 2015;60(2):333–338. 10.1007/s10620-014-3346-5. 25190263 10.1007/s10620-014-3346-5 PMC4385713

[12] Singh A, Dhaliwal AS, Singh S, et al. Awareness of nonalcoholic fatty liver disease is increasing but remains very low in a representative US cohort. Dig Dis Sci. 2020;65(4):978–986. 10.1007/s10620-019-05700-9. 31187324 10.1007/s10620-019-05700-9

[13] Wieland AC, Mettler P, McDermott MT, Crane LA, Cicutto LC, Bambha KM. Low awareness of nonalcoholic fatty liver disease among patients at high metabolic risk. J Clin Gastroenterol. 2015;49(1):e6–e10. 10.1097/MCG.0000000000000075. 24440943 10.1097/MCG.0000000000000075

[14] Cleveland ER, Ning H, Vos MB, et al. Low awareness of nonalcoholic fatty liver disease in a population-based cohort sample: the CARDIA study. J Gen Intern Med. 2019;34(12):2772–2778. 10.1007/s11606-019-05340-9. 31595464 10.1007/s11606-019-05340-9 PMC6854130

[15] Park J, MacLean MT, Lucas AM, et al. Exome-wide association analysis of CT imaging-derived hepatic fat in a medical biobank. Cell Rep Med. 2022;3(12):100855. 10.1016/j.xcrm.2022.100855. 10.1016/j.xcrm.2022.100855 PMC9798024 36513072

[16] MacLean MT, Jehangir Q, Vujkovic M, et al. Quantification of abdominal fat from computed tomography using deep learning and its association with electronic health records in an academic biobank. J Am Med Inform Assoc. 2021;28(6):1178–1187. 10.1093/jamia/ocaa342. 33576413 10.1093/jamia/ocaa342 PMC8661423

[17] Teng ML, Ng CH, Huang DQ, et al. Global incidence and prevalence of nonalcoholic fatty liver disease. Clin Mol Hepatol. 2023;29(Suppl):S32–S42. 10.3350/cmh.2022.0365. 36517002 10.3350/cmh.2022.0365 PMC10029957

[18] Elahi A, Cook TS. Artificial Intelligence Governance and Strategic Planning: How We Do It. Journal of the American College of Radiology. 2023;20(9):825–827. 10.1016/j.jacr.2023.06.017. 37453596 10.1016/j.jacr.2023.06.017

[19] Hammond WE. HL7--more than a communications standard. Stud Health Technol Inform. 2003;96:266–271. 15061555

[20] Kahn CE, Carrino JA, Flynn MJ, Peck DJ, Horii SC. DICOM and radiology: Past, present, and future. Journal of the American College of Radiology. 2007;4(9):652–657. 10.1016/j.jacr.2007.06.004. 17845973 10.1016/j.jacr.2007.06.004

[21] Kohli M, Alkasab T, Wang K, et al. Bending the artificial intelligence curve for radiology: Informatics tools from ACR and RSNA. Journal of the American College of Radiology. 2019;16(10):1464–1470. 10.1016/j.jacr.2019.06.009. 31319078 10.1016/j.jacr.2019.06.009

[22] Rubin DL, Kahn CE. Common data elements in radiology. Radiology. 2017;283(3):837–844. 10.1148/radiol.2016161553. 27831831 10.1148/radiol.2016161553

[23] Hamer OW, Aguirre DA, Casola G, Lavine JE, Woenckhaus M, Sirlin CB. Fatty liver: Imaging patterns and pitfalls. RadioGraphics. 2006;26(6):1637–1653. 10.1148/rg.266065004. 17102041 10.1148/rg.266065004

[24] Ma X, Holalkere N-S, Kambadakone R A, Mino-Kenudson M, Hahn PF, Sahani D V. Imaging-based quantification of hepatic fat: methods and clinical applications. Radiographics. 2009;29(5):1253–1277. 10.1148/rg.295085186. 19755595 10.1148/rg.295085186

[25] Boyce CJ, Pickhardt PJ, Kim DH, et al. Hepatic steatosis (fatty liver disease) in asymptomatic adults identified by unenhanced low-dose CT. American Journal of Roentgenology. 2010;194(3):623–628. 10.2214/AJR.09.2590. 20173137 10.2214/AJR.09.2590

[26] Kodama Y, Ng CS, Wu TT, et al. Comparison of CT methods for determining the fat content of the liver. American Journal of Roentgenology. 2007;188(5):1307–1312. 10.2214/AJR.06.0992. 17449775 10.2214/AJR.06.0992

[27] Kalshabay Y, Zholdybay Z, Di Martino M, et al. CT volume analysis in living donor liver transplantation: accuracy of three different approaches. Insights Imaging. 2023;14(1):82. 10.1186/s13244-023-01431-8. 37184628 10.1186/s13244-023-01431-8 PMC10185718

[28] Haberal M, Bayramoglu M, Kirnap M, Coskun M, Haberal M. Manual computed tomography liver volumetry. Transplantation. 2018;102(Supplement 7):S898. 10.1097/01.tp.0000543995.73966.e6.

[29] Hori M, Suzuki K, Epstein ML, Baron RL. Computed tomography liver volumetry using 3-dimensional image data in living donor liver transplantation: effects of the slice thickness on the volume calculation. Liver Transpl. 2011;17(12):1427–1436. 10.1002/lt.22419. 21850689 10.1002/lt.22419 PMC3226887

[30] U.S. Department of Health and Human Services Office for Civil Rights. https://ocrportal.hhs.gov/ocr/breach/breach_report.jsf. Accessed December 19, 2023.

[31] Gibney BT, Roberts JM, D’Ortenzio RM, et al. Preventing and mitigating radiology system failures: a guide to disaster planning. RadioGraphics. 2021;41(7):2111–2126. 10.1148/rg.2021210083. 34723695 10.1148/rg.2021210083

[32] Barker M, Chue Hong NP, Katz DS, et al. Introducing the FAIR principles for research software. Sci Data. 2022;9(1):622. 10.1038/s41597-022-01710-x. 36241754 10.1038/s41597-022-01710-x PMC9562067

[33] Wilkinson MD, Dumontier M, Aalbersberg IjJ, et al. The FAIR guiding principles for scientific data management and stewardship. Sci Data. 2016;3(1):160018. 10.1038/sdata.2016.18. 10.1038/sdata.2016.18 PMC4792175 26978244

[34] Ravi N, Chaturvedi P, Huerta EA, et al. FAIR principles for AI models with a practical application for accelerated high energy diffraction microscopy. Sci Data. 2022;9(1):657. 10.1038/s41597-022-01712-9. 36357431 10.1038/s41597-022-01712-9 PMC9649764

[35] Geis JR, Brady AP, Wu CC, et al. Ethics of artificial intelligence in radiology: Summary of the Joint European and North American multisociety statement. Radiology. 2019;293(2):436–440. 10.1148/radiol.2019191586. 31573399 10.1148/radiol.2019191586

[36] Seyyed-Kalantari L, Zhang H, McDermott MBA, Chen IY, Ghassemi M. Underdiagnosis bias of artificial intelligence algorithms applied to chest radiographs in under-served patient populations. Nat Med. 2021;27(12):2176–2182. 10.1038/s41591-021-01595-0. 34893776 10.1038/s41591-021-01595-0 PMC8674135

[37] FDA approves first treatment for patients with liver scarring due to fatty liver disease. https://www.fda.gov/news-events/press-announcements/fda-approves-first-treatment-patients-liver-scarring-due-fatty-liver-disease. 2014.

[38] Pickhardt PJ, Summers RM, Garrett JW, et al. Opportunistic screening: Radiology scientific expert panel. Radiology. 2023;307(5):e222044. 10.1148/radiol.222044. 37219444 10.1148/radiol.222044 PMC10315516

[39] Rusk N. The UK Biobank. Nat Methods. 2018;15(12):1001–1001. 10.1038/s41592-018-0245-2. 30504882 10.1038/s41592-018-0245-2

