# Supplementary figures and images for: A Cloud-Based System for Automated AI Image Analysis and Reporting
Source: J Imaging Inform Med. 2024 Jul 31;38(1):368–79. doi: 10.1007/s10278-024-01200-z (PMC11811354; doi:10.1007/s10278-024-01200-z)

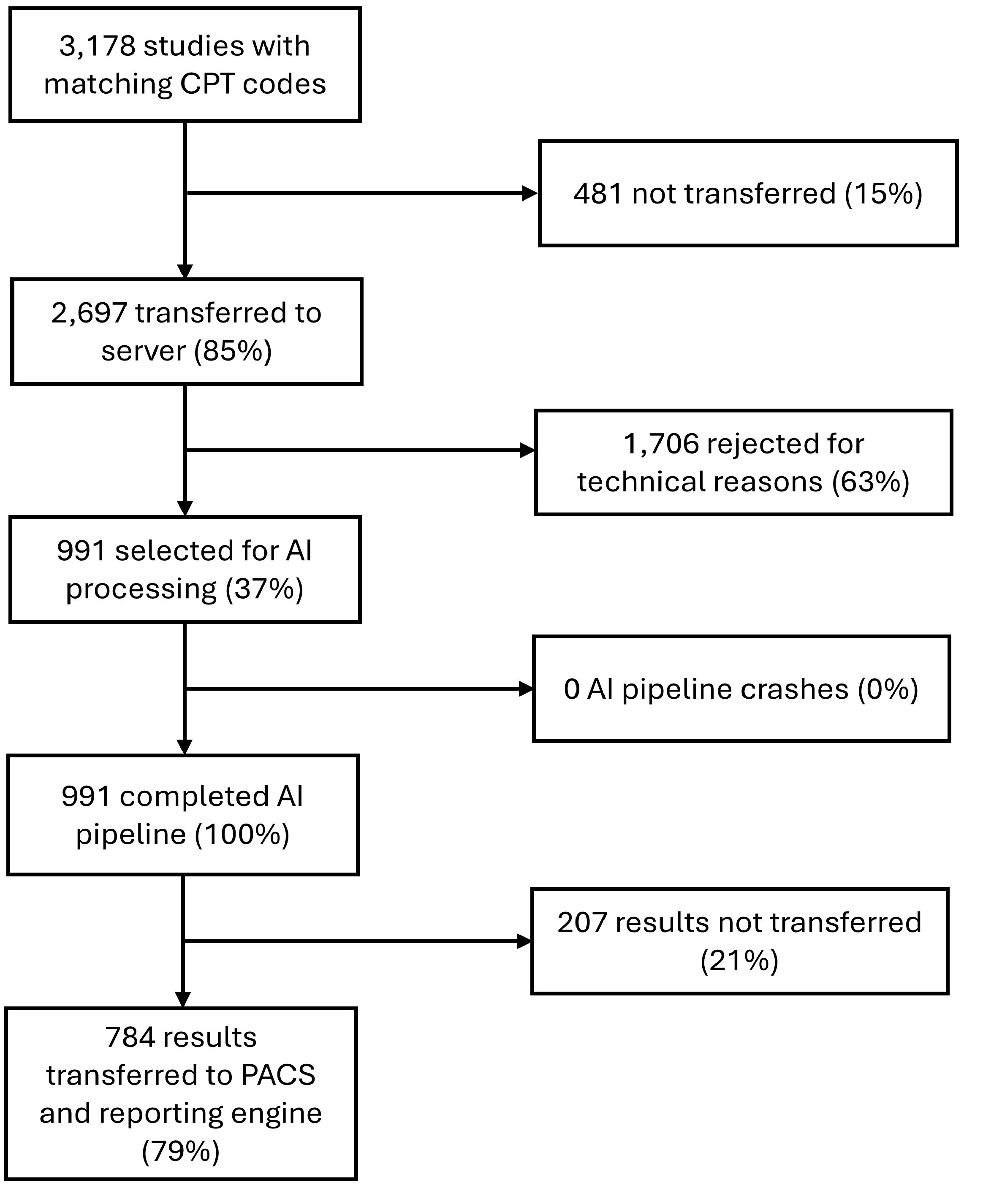

Supplement: Supplementary file 1 — Supplementary file1 (TIF 224 KB) [file 10278_2024_1200_MOESM1_ESM.tif]

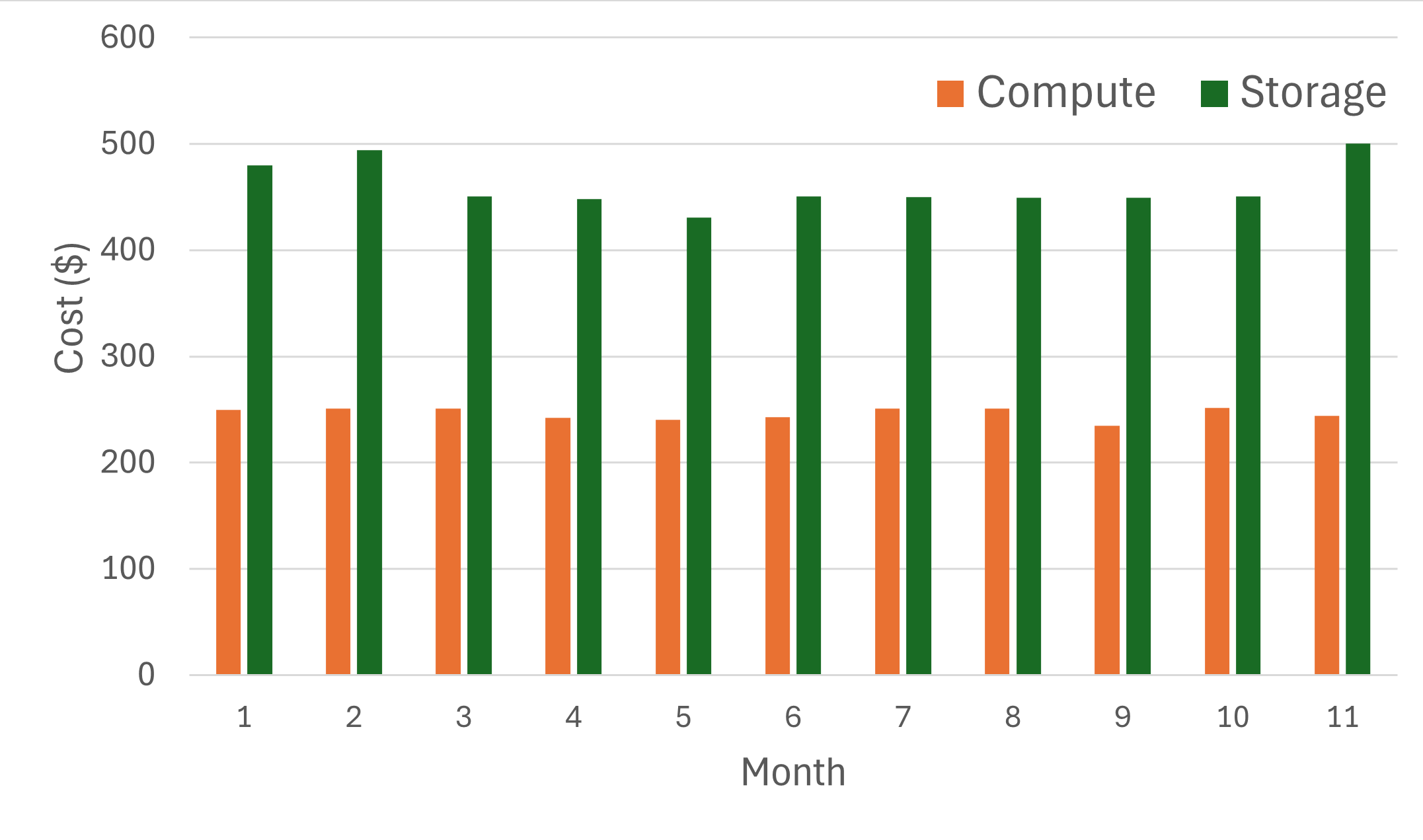

Supplement: Supplementary file 2 — Supplementary file2 (TIF 344 KB) [file 10278_2024_1200_MOESM2_ESM.tif]
