# Supplementary material for: A Cloud-Based System for Automated AI Image Analysis and Reporting
Source: J Imaging Inform Med. 2024 Jul 31;38(1):368–79. doi: 10.1007/s10278-024-01200-z (PMC11811354; doi:10.1007/s10278-024-01200-z)
Supplement: Supplementary file 3 — Supplementary file3 (DOCX 18.9 KB) [file 10278_2024_1200_MOESM3_ESM.docx]

**Supplementary Data Table 1**

The different scanner types and sites included in this study are summarized below:

| Site | Scanner |
| --- | --- |
| Site 1 | SOMATOM Definition AS+ |
| Site 2 | Biograph64_mCT 3R |
| Site 3 | SOMATOM Definition AS+ |
| Site 3 | Sensation 64 |
| Site 4 | SOMATOM Definition AS |
| Site 5 | SOMATOM go.Top |
| Site 6 | Revolution CT |
| Site 6 | SOMATOM Definition AS+ |
| Site 7 | LightSpeed VCT |
| Site 8 | SOMATOM Force |
| Site 8 | NAEOTOM Alpha |
| Site 8 | SOMATOM Definition Edge |
| Site 8 | SOMATOM Definition AS+ |
| Site 8 | SOMATOM Definition Flash |
| Site 8 | SOMATOM Drive |
| Site 9 | SOMATOM Definition AS+ |
| Site 10 | Spectral CT |
| Site 11 | Revolution CT |
| Site 11 | Revolution HD |
| Site 11 | SOMATOM Definition Edge |

**Supplementary Data Table 2**

The different common data elements (CDE) exported by the AI orchestrator is this study are summarized below. More specifics regarding each CDE can be found at https://www.radelement.org/

| CDE | Name |
| --- | --- |
| RDE1193 | Liver-spleen CT attenuation difference |
| RDE1194 | Liver noncontrast CT attenuation mean |
| RDE1195 | Liver noncontrast CT attenuation median |
| RDE1196 | Liver noncontrast CT attenuation maximum |
| RDE1197 | Liver noncontrast CT attenuation minimum |
| RDE1198 | Liver noncontrast CT attenuation skewness |
| RDE1199 | Liver elongation |
| RDE1200 | Liver Feret diameter |
| RDE1201 | Liver ellipsoid major axis length |
| RDE1202 | Liver ellipsoid minor axis length |
| RDE1213 | Spleen elongation |
| RDE1214 | Spleen Feret diameter |
| RDE1215 | Spleen ellipsoid major axis length |
| RDE1216 | Spleen ellipsoid minor axis length |
| RDE1217 | Spleen ellipsoid least axis length |
| RDE1218 | Spleen spherical radius |
| RDE1219 | Spleen roundness |
| RDE1220 | Liver volume |
| RDE1221 | Spleen volume |
| RDE1222 | Abdomen subcutaneous fat volume |

**Supplementary Figure 1**

Flowchart showing the number of studies at each step of the AI orchestrator workflow over a 60 day period of usage.

**Supplementary Figure 2**

Cloud computing costs over an 11 month period for the AI orchestrator. The 60 days analyzed for this study occurred over a period involving months 4-6.
